# Genomic Insights into Carbapenem-Resistant *Pseudomonas aeruginosa* (CRPA): Resistome and Virulome Analysis Beyond Carbapenemases

**DOI:** 10.3390/jcm15051683

**Published:** 2026-02-24

**Authors:** Marta Pantanella, Grazia Pavia, Nadia Marascio, Chiara Mazzei, Simona Gigliotti, Francesca Serapide, Alessandro Russo, Giovanni Matera, Angela Quirino

**Affiliations:** 1 Clinical Microbiology Unit, Department of Health Sciences, “Renato Dulbecco” University Hospital, 88100 Catanzaro, Italy; marta.pantanella@studenti.unicz.it (M.P.); graziapavia@unicz.it (G.P.); chiara.mazzei@studenti.unicz.it (C.M.); s.gigliotti@unicz.it (S.G.); mmatera@unicz.it (G.M.); quirino@unicz.it (A.Q.); 2 Infectious and Tropical Disease Unit, Department of Medical and Surgical Sciences, “Renato Dulbecco” University Hospital, 88100 Catanzaro, Italya.russo@unicz.it (A.R.)

**Keywords:** Carbapenem-resistant *Pseudomonas aeruginosa*, carbapenemases, resistome, virulome

## Abstract

**Background:** Carbapenem-resistant *Pseudomonas aeruginosa* (CRPA) has been added to the World Health Organization’s list as a high-priority pathogen for which new antibiotics are urgently needed. Herein, we investigated the association between resistance/virulence genes and high-risk CRPA clinical isolates by whole genome sequencing (WGS). **Methods**: Between 2019 and 2025, twenty-six CRPA strains from patients hospitalized in the “Renato Dulbecco” University Hospital were characterized. WGS analysis was performed using the next generation sequencing (NGS) technique. Multi-locus sequence typing (MLST) prediction was performed. Antibiotic resistance genes were detected using Antibiotic Resistance Gene-ANNOTation, Comprehensive Antibiotic Resistance Database, and ResFinder. Virulence genes were identified by the Virulence Factor Database. **Results**: The MLST analysis detected 14 different sequence types (ST). The 26 strains exhibited the same resistome profile: aac(3)-Ic, aphA15, catB7, catB10, cmlA, blaCARB, blaVIM-1, and tetG genes. The genes encoding enzymes involved in resistance to chloramphenicol and beta-lactams were found in all isolates using the three databases. Biofilm formation genes, metalloproteinase, chemotaxis, fimbriae, and pyoverdine were identified in all strains. Genes of the type III secretion system exoS, exoT, exoU, and exoY were found in 46.15%, 84.61%, 53.84%, and 84.61% of the strains, respectively. **Conclusions**: The analysis of the 26 clinical isolates showed high clonal heterogeneity, with a predominance of ST235, a high-risk clone associated with multiple resistances. Interestingly, cefiderocol resistance was carried by 4/8 isolates belonging to the ST235 strain. The surveillance based on resistome and virulome analysis could monitor the dynamic evolution of high priorityhigh-priority pathogens to guide clinical treatment and to adapt healthcare control measures, limiting their spread in the near future.

## 1. Introduction

Carbapenem-resistant *Pseudomonas aeruginosa* (CRPA) has been added to the World Health Organization (WHO) list as a high-priority pathogen for which new therapeutic options are urgently needed [1,2]. As a member of the ESKAPE group, including *Enterococcus faecium*, *Staphylococcus aureus*, *Klebsiella pneumoniae*, *Acinetobacter baumannii*, and *Enterobacter* spp., *P. aeruginosa* is recognized for its high intrinsic and potential acquired resistance and its prominent role in healthcare-associated infections (HAIs) [3].

*P. aeruginosa* is a ubiquitous opportunistic Gram-negative bacterium implicated in both acute and chronic diseases, especially in immunocompromised patients with underlying conditions such as chronic obstructive pulmonary disease (COPD), ventilator-associated pneumonia (VAP), cystic fibrosis (CF), or hematological disorders [4,5]. It is characterized by remarkable genomic plasticity and environmental adaptability, which enable it to colonize several ecological niches, including aquatic, soil, and human-associated settings [6]. The *P. aeruginosa* genome, spanning 5.5–7.0 Mb, is larger than that of many other bacterial pathogens and encodes a broad repertoire of virulence and resistance determinants, collectively referred to as the virulome and resistome [7].

Globally, *P. aeruginosa* infections are associated with more than 300,000 deaths each year, with an expanding dissemination of CRPA strains [8]. In Europe, the incidence of invasive *P. aeruginosa* isolates increased by 6.4% between 2022 and 2023, highlighting a sustained upward trend in CRPA spread [9,10]. In Italy, 6510 CRPA isolates were reported, corresponding to an overall resistance rate of 8.5% in 2023 [10]. At the regional level, surveillance data from the Calabria Region showed a 16.4% CRPA prevalence, representing one of the most concerning epidemiological trends at the subnational level [11].

Carbapenem resistance in *P. aeruginosa* is a multifactorial and synergistic phenomenon driven by both enzymatic and non-enzymatic mechanisms [12,13]. These include the loss or structural alteration of porins, overproduction of β-lactam–inactivating enzymes, upregulation of multidrug efflux pumps, and acquisition of carbapenemase-encoding genes [12,13]. Carbapenemase production has significant clinical consequences, as it often confers resistance not only to carbapenems but also to other β-lactams, including several newer β-lactam/β-lactamase inhibitor combinations, potentially worsening clinical outcomes [14,15,16,17]. While carbapenemase has been a major driver of resistance in *Enterobacterales* [18], its contribution to CRPA is not yet fully defined. The loss of the outer membrane porin OprD, often in combination with efflux pump overexpression or AmpC β-lactamase hyperproduction, has been identified as the main mechanism responsible for carbapenem resistance in the absence of carbapenemase production [19,20].

However, in routine clinical diagnostics, most rapid assays for CRPA detection target only carbapenemase genes [21]. This approach does not account for the broad complexity of the *P. aeruginosa* resistome and virulome, limiting both outcome prediction and optimal clinical management [22,23]. Recently, whole genome sequencing (WGS) has emerged as a powerful tool for strain typing of multidrug-resistant (MDR) *P. aeruginosa*, with applications in both surveillance and outbreak investigations [24]. It provides comprehensive insights into the resistome, virulome, and sequence-based typing (STs) of the main circulating high-risk clones of clinical relevance [25], such as ST235, ST111, ST233, ST244, ST357, ST308, ST175, ST277, ST654, and ST298 [26,27].

The present study aimed to perform a comprehensive genomic characterization of clinical CRPA isolates circulating in our teaching hospital during the last six years, with a specific focus on the genetic determinants of antimicrobial resistance and virulence. To delineate MDR profiles, the resistome and virulome of selected clinically relevant CRPA strains were investigated using a WGS-based approach.

## 2. Materials and Methods

### 2.1. Study Design

**Figure 1:**
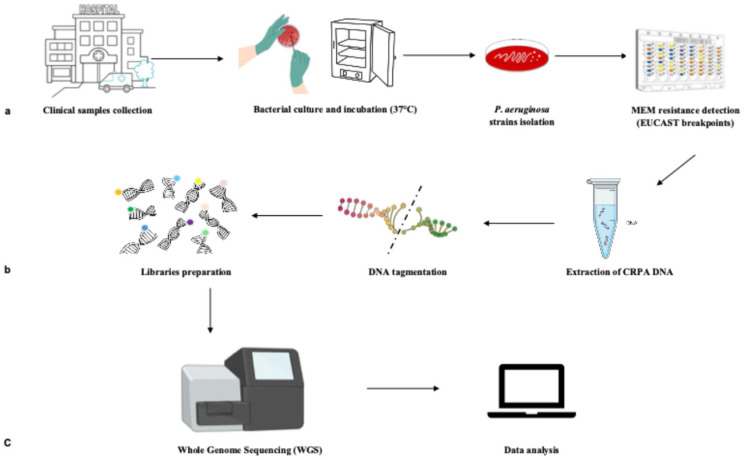
Steps of the study design. (**a**) *P. aeruginosa* strains were identified using routinely diagnosis from clinical samples collected at University Hospital. (**b**) Metagenomic analysis was applied to *P. aeruginosa* meropenem (MEM) resistant (CRPA) isolates. (**c**) Whole genome of CRPA strains was analyzed to detect resistance and virulence genes by WGS and specific software (data analysis).

Between 1 January 2019 and 30 June 2025, 1131 *P. aeruginosa* were collected from the patients admitted to the “Renato Dulbecco” University Hospital of Catanzaro, Southern Italy. The bacterial isolate from the first positive sample, showing phenotypic resistance to meropenem (MEM), was included in the study as CRPA. The patient’s cohort presented severe pneumonia, acute respiratory distress syndrome (ARDS), sepsis, or septic shock. CRPA strains were classified according to the source of isolation. Invasive isolates were obtained from sterile body sites, including blood, urine, bronchial aspirate (BAS), and bronchoalveolar lavage (BAL). Colonizing isolates were recovered from surveillance samples, such as throat swabs (TS), rectal swabs (RS), and wound swabs (WS). CRPA isolates from patients without selected clinical conditions or with incomplete clinical data were excluded from the study. All demographic and clinical data were collected in an anonymized database. To characterize the resistome and virulome of the clinical CRPA isolates, WGS and data analysis were performed (Figure 1).

### 2.2. Identification and Antimicrobial Susceptibility Testing

*P. aeruginosa* strains were cultured on MacConkey agar (bioMérieux, Craponne, France) and incubated aerobically at 37 °C for 18–24 h before further analyses. Isolate identification was performed using matrix-assisted laser desorption/ionization time-of-flight mass spectrometry (MALDI-TOF MS). Antimicrobial susceptibility testing (AST) was carried out using the automated VITEK^®^ 2 system (bioMérieux, Craponne, France) and the broth microdilution method (Sensititre; Thermo Fisher Scientific, Waltham, MA, USA). The following antimicrobial agents were tested: amikacin (AK), cefepime (FEP), colistin (CS), MEM, piperacillin–tazobactam (TZP), imipenem–relebactam (IMR), meropenem–vaborbactam (MEV), ceftazidime–avibactam (CAZ-AVI), ceftolozane–tazobactam (C/T), and cefiderocol (FDC). Interpretation of susceptibility results was performed according to the European Committee on Antimicrobial Susceptibility Testing (EUCAST) clinical breakpoints, version 15.0 [28]. In addition, cefiderocol susceptibility was assessed by the disk diffusion method using 30-µg disks (Liofilchem S.r.l., Roseto degli Abruzzi, Italy), following EUCAST recommendations [28].

### 2.3. WGS Approach

Genomic DNA was extracted using the QIAamp^®^ DNA Mini Kit (QIAGEN, Hilden, Germany), according to the manufacturer’s instructions. DNA concentration was determined using the Qubit™ dsDNA High Sensitivity Assay Kit (Invitrogen, Carlsbad, CA, USA) to assess input DNA quantity. Whole-genome sequencing (WGS) libraries were prepared using the Illumina DNA Prep protocol and sequenced on the Illumina MiSeq platform (Illumina, San Diego, CA, USA). An input of 100 ng of genomic DNA per sample was used for library preparation. Indexing was performed using the Illumina DNA/RNA UD Indexes Set A (Illumina, San Diego, CA, USA). Library concentration and quality were evaluated using the Qubit™ dsDNA High Sensitivity Assay Kit (Invitrogen, Carlsbad, CA, USA) and the Agilent^®^ High Sensitivity DNA Kit (Agilent, Santa Clara, CA, USA). Library denaturation and dilution were carried out according to the manufacturer’s instructions, at a final loading concentration of 12 pM. Sequencing was performed using MiSeq Reagent Kits v3 (Illumina, San Diego, CA, USA).

### 2.4. Data Analysis

The high-throughput data were analyzed using BaseSpace software v7.37.0 (Illumina, Inc., San Diego, CA, USA). The SRST2 version 1.1.0 app was applied to assign virulence and multilocus sequence typing (MLST) genes. Antimicrobial resistance determinants were predicted by Antibiotic Resistance Gene-ANNOTation (ARG-ANNOT), Comprehensive Antibiotic Resistance Database (CARD), and ResFinder. Virulence genes were identified using the Virulence Factor Database (VFDB). The PlasmidFinder tool was used to identify plasmid replicon genes.

### 2.5. Statistical Analysis

Fisher’s exact test (GraphPad Prism v10.4.1, GraphPad Software Inc., Boston, MA, USA) was performed to compare the presence of specific virulence genes between CRPA invasive and colonizing strains. A *p*-value of less than 0.05 was considered statistically significant.

## 3. Results

### 3.1. Patient’s Cohort Characteristics

The 26 patients with culture-confirmed CRPA colonization or infection were 14 males (53.8%) and 12 females (46.2%), with a median age of 64.5 years (range 18–88). Most patients were admitted to the intensive care (ICU, 15/26, 57.7%), infectious diseases (IDU, 3/26, 12%), oncology (ONCO, 2/26, 8%), and pneumology (PNEU, 2/26, 8%) wards (Table 1).

Sepsis (10/26, 38%) and severe pneumonia (9/26, 35%) emerged as the most frequent clinical presentations at admission, often complicated by the development of ARDS. Overall, ARDS was documented in 11 patients (42%), occurring either as an isolated condition or in combination with sepsis or septic shock. Septic shock was observed in six patients (23%), all of whom required admission to the ICU. Concerning underlying diseases, metabolic disorders were the most prevalent primary conditions (6/26, 23%), followed by chronic kidney disease (CKD) and infectious diseases, each reported in 19.2% of cases (5/26). Cardiovascular and cerebrovascular diseases were identified in four patients (15%), autoimmune or inflammatory conditions in three patients (12%), while neurological, oncological, and chronic pulmonary diseases were each present in two patients (8%). A majority of patients exhibited multiple comorbidities, frequently overlapping. Cardio-metabolic conditions were the most common comorbidity (7/26, 27%), followed by renal-related diseases and endocrine or autoimmune disorders (4/26, 15.4%). Neurological or psychiatric comorbidities were relatively uncommon, occurring in only two patients (7.7%). Additionally, in-hospital mortality (9/26, 34.6%) was markedly higher among patients admitted to the ICU (7/15, 46.7%) compared with those managed in non-ICU wards (2/11, 18.2%) and was strongly associated with severe clinical presentations. In particular, 7 of 11 ICU patients (63.6%) presenting with septic shock and/or ARDS died during hospitalization, whereas only 2 of 15 patients (13.3%) without these complications experienced a fatal outcome (Table 1).

### 3.2. CRPA Isolates: Phenotypic Features and Circulating Sequence Types (STs)

This study characterized 26 unique CRPA clinical isolates, with a 2.3% (26/1131) prevalence of *P. aeruginosa* collected during the time span of six years. The antimicrobial drug susceptibility test results were detailed in Table 2.

Based on the source of isolation, 14 isolates (54%) were invasive, while 12 isolates (46%) were colonizing strains. At the time of isolation, 11 patients (42%) received antimicrobial therapy. All isolates showed an antimicrobial susceptibility profile of resistance (R or I) to MEM and TZP, confirming their carbapenem-resistant phenotype. High rates of resistance were also observed for cefepime (22/26, 85%) and amikacin (3/26, 11%), whereas colistin retained activity against the majority of strains (24/26, 92%). In vitro susceptibility to imipenem/relebactam and meropenem/vaborbactam was limited to four (15%) and two (8%) isolates, respectively. Newer β-lactam/β-lactamase inhibitor combinations demonstrated variable activity: ceftazidime/avibactam and ceftolozane/tazobactam were active against 14 (54%) and 15 (58%) of total isolates, respectively. On the other hand, cefiderocol showed the highest activity; 22/26 (85%) isolates remained susceptible.

**Figure 2:**
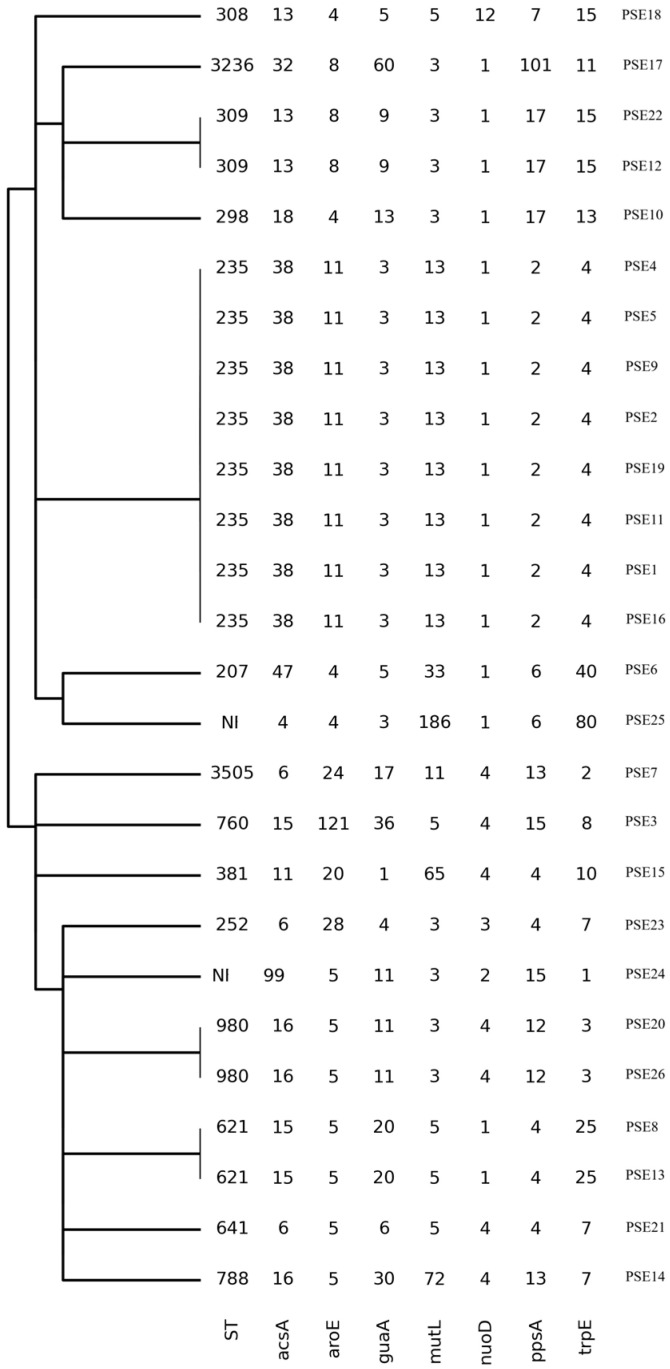
Illustration of MLST results. The dendrogram showed genetic distance among the 26 STs. Two out of 26 ST were not identifiable (NI).

The MLST analysis based on seven housekeeping genes—acetyl-coenzyme A synthetase (*acsA*), shikimate dehydrogenase (*aroE*), GMP synthase (*guaA*), DNA mismatch repair protein (*mutL*), NADH dehydrogenase I chain C, D (*nuoD*), phosphoenolpyruvate synthase (*ppsA*), and anthralite synthetase component I (*trpE*)—revealed a heterogeneous distribution of STs. The high-risk clone ST235 was the most prevalent lineage, identified in eight isolates (31%), and was recovered from both invasive and colonizing specimens. Other sequence types were sporadically identified, including ST207, ST252, ST298, ST308, ST309, ST381, ST621, ST641, ST760, ST788, and ST980. Two isolates (2/26, 7.7%) could not be assigned to a known ST (Figure 2).

### 3.3. Resistome and Virulome of CRPA Strains

Resistome analysis was conducted by three databases: ARG-ANNOT, CARD, and ResFinder. The following genes were identified using the ARG-ANNOT database: *aac*(3)-Ic, *aac*Aad, *aph*(3)-IIb, and *aph*A15 involved in aminoglycoside resistance; *bla*_CARB_, *bla*_GES_, *bla*_OXA-50_, and *bla*_VIM-1_ implicated in beta-lactam antibiotic resistance; *cat*B10, *cat*B7, and *cml*A genes for chloramphenicol resistance; *tet*G and *sul*I1616 involved in resistance to tetracycline and sulphonamides, respectively. All strains harbored *bla*_OXA-50_ genes, except for one isolate (PSE1). The most prevalent resistance genes were: *aph*(3)-IIb, *bla*_OXA-50_, and *cat*B7.

The following genes were found after performing an analysis on the CARD dataset: *aac*(3)-Ic, *aac*(6)-Ib, *ant*(3), *aph*(3), and *aph*A15 aminoglycoside resistance genes; *cat*B7, *cat*B10, and *cml*A involved in chloramphenicol resistance; *bla*_CARB_, *bla*_GES-7_, *bla*_GES-17_, *bla*_OXA-50_, *bla*_OXA-488_, *bla*_OXA-486_, *bla*_PDC_, and *bla*_VIM-1_ beta-lactam resistance determinants; and *crp*P and *tet*G associated with fluoroquinolone and tetracycline resistance, respectively. The main detected resistance gene was *bla*_PDC_.

Several ARGs were identified by ResFinder: *aac*(3)-Ic, *aac*(6)-Ic, *ant*(3), *aph*(3)-IIb, and *aph*A15. All of them were aminoglycoside resistance genes; *cat*B7, *cat*B10, and *cml*A, which are involved in chloramphenicol resistance; *bla*_CARB_, *bla*_GES-7_, *bla*_OXA-50_, *bla*_OXA-488_, *bla*_PAO_, and *bla*_VIM-1_, which are genes encoding beta-lactam resistance; and *crp*P, *fos*A, and *tet*G, which are involved in resistance to fluoroquinolones, fosfomycin, and tetracycline, respectively. High prevalence of *bla*_PAO_ and *fos*A1 genes was observed among CRPA strains. A few discordances were detected for amikacin, where the presence of at least one gene did not match the phenotypic amikacin resistance profile on antimicrobial susceptibility testing.

**Figure 3:**
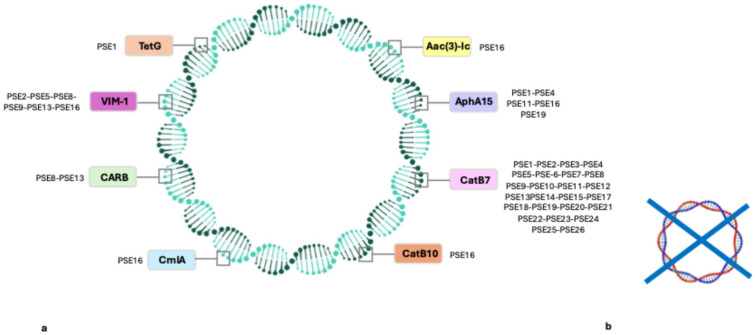
(**a**) Schematic representation of chromosomal DNA (green double helix) and resistance genes (colored boxes) detected on CRPA strains by the combination of ARG-ANNOT, CARD, and ResFinder databases. Every detected resistance gene (TetG, VIM-1, CARB, CmIA, CatB10, CatB7, AphA15 and Aac(3)-Ic) was associated with the CRPA isolate ID (identified as PSE- plus isolate number as reported in Table 2). (**b**) No resistance genes (symbol x) were found on the plasmid DNA (red and blue double helix) of CRPA strains.

The strains exhibited the same resistance gene profile according to all three databases (Figure 3).

**Figure 4:**
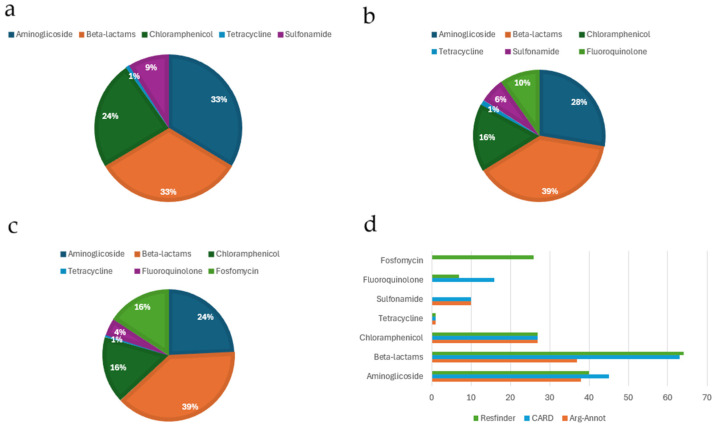
Prevalence (%) of resistance genes detected in clinical isolates according to different antibiotic classes using (**a**) ARG-ANNOT, (**b**) CARD, (**c**) ResFinder, and (**d**) the combination of the three databases.

Interestingly, different resistance pattern of antibiotic classes was detected using the three databases (Figure 4). In particular, genes encoding enzymes involved in resistance to chloramphenicol and beta-lactams were found in all isolates by all databases. The fosfomycin resistance gene was detected by the ResFinder, while sulfonamide resistance genes were detected using ARG-ANNOT and CARD (Figure 4).

All isolated strains displayed multiple virulence factors (Supplementary Table S1). Biofilm formation genes, *alg*A-B-C-D-E-F-G-I-J-K-L-P-Q-R-U-W-X-Z, were present in all strains. The genes for the metalloproteinase (*apr*A), chemotaxis (*che*Y-Z, *chp*A-B-C-D-E), fimbriae (*cup*A1-A2-A3-A4-A5-B1-B2-B3-B4-B5-B6-C2-C3), except *cup*C1 and esterase (*est*A), were detected in all isolates. Genes of the effectors of the type III secretion system *exo*S (42% of colonized and 50% of infected patients), *exo*T (92% of colonized and 79% of infected patients), *exo*U (58,3% of colonized and 50% of infected patients), and *exo*Y (83,3% of colonized and 86% of infected patients) were variably found. A complete set of genes for the transcriptional regulator (*exs*CEBA), component of the fibril functional amyloid (*fap*ABCDEF), type IV fimbriae (*fim*T, *fim*U), flagellar protein (*flg*A-B-C-D-E-F-G-H-I-J), and pyoverdine (pvdA-E-F-G-H-L-N) was revealed. Interestingly, only the PSE15 strain showed the exclusive presence of 15 genes: *his*F2, *his*H2, *wbp*ABDEGHIJKL, and *wz*XYZ. Significantly, by Fisher’s exact test (*p*-value < 0.0001), 60% of invasive strains displayed the virulence genes involved in the pyoverdine biosynthesis (fpvI, pvdJ), sigma regulation (fpvR, pvdS), fimbrial biosynthesis (pilC), and acetylation of hydroxy ornithine (pvdY).

## 4. Discussion

This study reported the first comprehensive characterization of CRPA isolated from clinical samples in the Calabria Region. We provide an integrated clinical, phenotypic, and genomic analysis of CRPA isolates recovered from a heterogeneous cohort of hospitalized patients. Our results offer new insights into the epidemiology and pathogenic potential of this high-risk strain in a healthcare setting. Resistome and virulome analyses demonstrated that all our isolates revealed a broad array of resistance genes conferring resistance to multiple antibiotic classes and an extensive repertoire of virulence determinants (326 genes).

*P. aeruginosa* is recognized as one of the most frequent causes of HAIs, particularly in critically ill patients and in those requiring invasive devices such as mechanical ventilation [29]. Its prevalence among HAIs has been estimated at approximately 7–8%, with higher rates reported in ICUs, where severe pneumonia, bloodstream infections, and sepsis are most commonly observed [30]. Consistent with these observations, the majority of patients in our cohort were admitted to the ICU (57.7%) and presented with severe clinical syndromes, including sepsis (38%), severe pneumonia (35%), ARDS (42%), and septic shock (23%). These conditions were strongly associated with adverse outcomes, as reflected by an overall in-hospital mortality rate of 34.6%, which increased to 46.7% among ICU patients. Mortality was particularly high in patients presenting with septic shock and/or ARDS, highlighting the synergistic impact of immunodeficiency, disease severity, and CRPA infection [30]. The high prevalence of multiple comorbidities in our study cohort, especially cardio-metabolic disorders, chronic kidney disease, and immunological or infectious conditions, such as HIV or HCV infection, further supports current knowledge that CRPA primarily affects vulnerable hosts with limited immunological response [31].

Indeed, this high-risk pathogen has several resistance determinants and virulence factors that enable it to adapt to different environments [6]. In particular, carbapenem resistance in *P. aeruginosa* is due to several key mechanisms. The first involves increased efflux of antibiotics, or, additionally, AmpC β-lactamase overproduction and loss of the OprD outer membrane porin. Another mechanism, more frequently observed, involves the acquisition and production of carbapenemases [32]. The metallo-β-lactamases, particularly the VIM family, are the most prevalent carbapenemases worldwide, followed by IMP and NDM types [33]. Next-generation sequencing enhanced new approaches for surveillance and control of these pathogens [34]. WGS enabled a more in-depth bioinformatic analysis of resistome and virulome to assess the potential clinical impact of local isolates.

All CRPA strains included in our study displayed high rates of phenotypic antimicrobial resistance, particularly for cefepime (85%), while susceptibility to aminoglycosides was partially retained, with amikacin resistance detected in a minority of isolates. Colistin remained active against most strains (92%), although the identification of a colistin-resistant isolate is clinically relevant, given the role of colistin as a last-resort therapy [35]. Susceptibility to newer β-lactam/β-lactamase inhibitor combinations was limited, with imipenem/relebactam and meropenem/vaborbactam showing activity in only 15% and 8% of isolates, respectively. In contrast, ceftazidime/avibactam and ceftolozane/tazobactam retained activity against approximately half of the strains, while cefiderocol demonstrated the highest in vitro efficacy (85%). Nevertheless, the detection of cefiderocol resistance, particularly among high-risk ST clones, raises concerns regarding the future effectiveness of this drug [36].

MLST analysis revealed substantial clonal heterogeneity, with both invasive (54%) and colonizing (46%) isolates distributed across multiple STs (ST207, ST235, ST252, ST298, ST308, ST309, ST381, ST621, ST641, ST760, ST788, and ST980). Despite this diversity, the high-risk clone ST235 emerged as the predominant lineage, accounting for 31% of isolates and persisting throughout the entire study period, in line with global dissemination data of ST235 and correlating with our MDR and extensively drug-resistant (XDR) strains [37]. Indeed, ST235 exhibits a highly virulent phenotype, associated with elevated mortality, which is likely attributable to the production of the ExoU cytotoxin [38]. All our strains with ST235 had the *exo*U gene, but only patients #11 and #1 had an adverse outcome (Table 1). Interestingly, PSE1, PSE4, PSE11, PSE16, and PSE19 isolates possessed the highest number of resistance genes and, at the same time, were ST235 clones. Particularly relevant is the evidence of resistance to cefiderocol, a latest-generation cephalosporin [39,40], which is present in all four isolates belonging to the high-risk ST235 clone. Furthermore, for strains PSE2, PSE5, PSE9, and PSE16, the presence of the VIM-1 gene was associated with the high-risk clone ST235 [41]. In our cohort, patient #10, infected by ST298, and patient #18, colonized by the ST308 high-risk clone, died. Also, the colistin-resistant strain isolated from patient #6 (PSE22), which belonged to ST309, was classified as a high-risk clone [42,43]. The ST381 strain was isolated from the BAL of patient #7. Recently, ST381 was found to be a clone that exhibits resistance to carbapenems, which is consistent with our results [44]. The MLST revealed new allele combinations of clones: ST760, ST3236, and ST3505. Among these, only patient #3 (infected by ST760) had a fatal outcome.

Core β-lactamases, including blaOXA-50, blaPDC, and blaPAO, were detected in nearly all isolates, reflecting intrinsic resistance mechanisms of *P. aeruginosa* [3]. Acquired carbapenemase genes were less frequent; blaVIM-1 was identified in only three isolates, predominantly associated with ST235, while blaGES variants were detected in four isolates belonging to the same high-risk clone. Aminoglycoside resistance genes, such as aph(3)-IIb and aac variants, were widely distributed, although discordances between genotypic findings and phenotypic susceptibility, particularly for amikacin, were observed. These discrepancies highlight that resistance expression in *P. aeruginosa* is multifactorial and cannot be reliably predicted based solely on gene presence [45]. The universal detection of fosA1 and the high prevalence of blaPAO further emphasize the complex intrinsic resistome of CRPA strains [3]. Notably, the blaVIM1 gene was found only in six isolates. The absence of common carbapenemase genes in our isolates suggests other variations in resistance mechanisms. In contrast, for *bla*_PAO_ and *bla*_PDC_ cephalosporinases, whose genes were found in all isolates, only 65.4% showed resistance to cephalosporins. In particular, the presence of PDC is in line with what was reported in the study by Mack [46]. The *bla*_GES_ gene was present only in PSE1, PSE4, PSE11, and PSE19 strains. Indeed, our results were in line with the literature data [47], and these four isolates are associated with high-risk ST235 clones. In *P. aeruginosa* ST235, the tetG gene was harbored [48]. Eight out of ten strains displayed the co-resistance to ceftazidime–avibactam and ceftolozane–tazobactam related to the presence of *bla*_OXA-488_; strains #17 and #18, carrying this gene, were sensitive to both antibiotics [49]. CARD is one of the most detailed, extensive, and comprehensive databases [50]. Indeed, even our CARD dataset includes several variants. There are two *bla*_OXA-50_/bla_VIM-1_ and four *bla*_OXA-488_/*bla*_VIM-1_ genes. A variety of GES genes were detected: GES-7 (8%—2/26) and GES-17 (8%—2/26).

A comparative analysis of gene distribution was performed between strains responsible for patient colonization (n = 12) and infection (n = 14). The virulence genes involved in biofilm or pyoverdin formation [51] were found in all strains. Furthermore, all isolates have highly conserved core genes linked to key functions such as flagellar motility (*flg*, *fli*) [52], adhesion (*cup*) [53], and type III and IV secretion systems (*psc*) [54]. The differing distribution of effectors of the type III secretion system (such as *exo*S, *exo*T, *exo*U, and *exo*Y) underlines the variability of pathogenic potential between strains, with *exo*U particularly relevant for clinical prognosis [55]. Other genes associated with polysaccharide modification and processing also showed comparable frequencies between the two groups, with no marked differences in prevalence. Even though the gene presence/absence profiles were largely overlapping between colonizing and invasive isolates, with a high degree of genetic similarity, six virulence genes involved in the pyoverdine biosynthesis, sigma regulation, fimbrial biosynthesis, and acetylation of hydroxy ornithine, were detected solely in invasive strains.

## 5. Conclusions

The resistome and virulome of CRPA comprise a wide array of resistance and virulence determinants belonging to multiple functional categories, reflecting the remarkable adaptive capacity of this pathogen. To our knowledge, no published papers have reported resistome and virulome analyses of CRPA clinical isolates in Southern Italy. Despite the small sample size, we have provided a comprehensive and representative overview of circulating strains in the Calabria Region, increasing phenotypic and genomic information within the Italian landscape. Even if the ongoing surveillance could improve the robustness of the results, our preliminary data established for the first time the temporal trends, such as the increase in CRPA isolation from 2019 (1/26) to 2024 (11/26), and the specific virulence patterns (fpvI, pvdJ, fpvR, pvdS, pilC, and pvdY genes) detected only in invasive strains. The analysis of 26 clinical isolates over the time span of six years revealed a high degree of clonal heterogeneity, with a predominance of the globally disseminated high-risk clone ST235. As previously reported, this clone was associated with enhanced virulence and multidrug resistance, including reduced susceptibility to last-line agents, such as cefiderocol. The integrated clinical, phenotypic, and WGS genomic analyses as surveillance approaches could monitor the dynamic evolution of molecular mechanisms to guide clinical treatment and to help adapt the healthcare control measures, limiting the spread of high-priority pathogens locally and consequently beyond regional borders in the near future.

## Figures and Tables

**Table 1:** Clinical characteristics of the patients included in the study cohort.

Patient ID	Clinical Condition	Primary Disease	Comorbidity	Death
PZ1	Severe Pneumonia; ARDS	CeVDs	None	Yes
PZ2	Sepsis	DM2	CaVDs; DLP	No
PZ3	Severe Pneumonia	Parkinson’s disease	Hypothyroidism; Psychiatric disease	Yes
PZ4	ARDS	SSc	HF	No
PZ5	Sepsis; ARDS	SO	None	No
PZ6	Sepsis; ARDS	COVID-19	None	Yes
PZ7	Sepsis; ARDS	COVID-19	None	No
PZ8	Sepsis	BICa	DM2; SO; DLP; CKD	No
PZ9	Sepsis; ARDS	Epilepsy	Tetraparesis	No
PZ10	Septic shock; ARDS	COVID-19	COPD	Yes
PZ11	Severe Pneumonia	DM2	None	Yes
PZ12	Severe Pneumonia	HIV	Hypertension, PML	No
PZ13	Sepsis	CaVDs	DLP	No
PZ14	ARDS	None	None	No
PZ15	Severe Pneumonia	ACOS	None	No
PZ16	Severe Pneumonia	GPA	Hypertension; CKD on dialysis	No
PZ17	Severe Pneumonia	CHC	None	No
PZ18	Septic shock; ARDS	CKD	None	Yes
PZ19	Severe Pneumonia	CaVDs	None	No
PZ20	Septic shock; ARDS	CKD on dialysis	Hypothyroidism; SO	No
PZ21	Sepsis	DM2	Hypertension; DLP	No
PZ22	Severe Pneumonia	SRS	None	No
PZ23	Sepsis	BrCa	None	No
PZ24	Septic shock; ARDS	CKD on dialysis	None	Yes
PZ25	Septic shock	CM	None	Yes
PZ26	Sepsis	DM2	CKD	No

ARDS = acute respiratory distress syndrome; HIV = human immunodeficiency virus infection; PML = progressive multifocal leukoencephalopathy; COVID-19 = coronavirus disease 2019; COPD = chronic obstructive pulmonary disease; SSc = systemic sclerosis; HF = hearth failure; ACOS = asthma-COPD overlap syndrome; GPA = granulomatosis with polyangiitis; CKD = chronic kidney disease; CHC = chronic hepatitis C; DM2 = type 2 diabetes mellitus; CaVDs = cardiovascular diseases; CM = compressive myelopathy; DLP = dyslipidemia; SRS = Silver–Russell syndrome; CeVDs = cerebrovascular diseases; SO = severe obesity; BrCa = breast cancer; BlCa = bladder cancer.

**Table 2:** Phenotypic features of CRPA strains.

Isolate ID	Date	Source	Phenotypic Antimicrobial Susceptibility Testing (EUCAST Guidelines)									
			AK	FEP	CS	MEM	TZP	IMR	MEV	CAZ-AVI	C/T	FDC
PSE1	2019	RS	R	R	S	R	R	R	R	R	R	S
PSE2	2020	US	S	R	S	R	R	R	R	R	R	S
PSE3	2022	BAS	S	R	S	R	R	R	R	S	S	S
PSE4	2022	BAL	R	R	S	R	R	R	R	R	R	R
PSE5	2022	BC	S	R	S	R	R	R	R	R	R	R
PSE6	2022	BC	S	I	S	R	I	S	S	S	S	S
PSE7	2022	US	S	R	S	R	R	R	S	S	S	S
PSE8	2022	US	S	R	S	R	R	R	R	R	R	S
PSE9	2022	WS	S	R	S	R	R	R	R	R	R	S
PSE10	2023	BAS	S	I	S	R	R	S	R	S	S	S
PSE11	2023	TS	R	R	S	R	R	S	R	R	R	R
PSE12	2023	BAS	S	I	S	R	R	S	R	S	S	S
PSE13	2023	US	S	R	S	R	R	R	R	R	R	S
PSE14	2024	BAS	S	R	R	R	R	S	S	S	S	S
PSE15	2024	BAL	S	R	S	R	R	S	R	R	R	S
PSE16	2024	BAL	S	R	S	R	R	R	R	R	R	S
PSE17	2024	TS	S	R	S	R	R	S	S	S	S	S
PSE18	2024	TS	S	I	S	R	I	R	R	S	S	S
PSE19	2024	TS	S	R	S	R	R	S	S	R	R	R
PSE20	2024	TS	S	I	S	R	R	S	R	S	S	S
PSE21	2024	WS	S	R	S	R	R	S	R	R	S	S
PSE22	2024	WS	S	I	R	R	R	R	R	S	S	S
PSE23	2024	BC	S	I	S	R	R	R	S	S	S	S
PSE24	2024	TS	S	I	S	R	R	S	S	S	S	S
PSE25	2025	TS	S	R	S	R	R	S	R	S	S	S
PSE26	2025	WS	S	I	S	R	I	S	S	S	S	S

PSE = *Pseudomonas aeruginosa*; BAS = bronchial aspirate; BAL = bronchial lavage; TS = Throat Swab; WS = wound swab; RS = rectal swab; BC = blood culture; US = urine sample; AK = amikacin; FEP = cefepime; CS = colistin; MEM = meropenem; TZP = piperacillin/tazobactam; IMR = imipenem/relebactam; MEV = meropenem/vaborbactam; CAZ-AVI = ceftazidime/avibactam; C/T = ceftalozane/tazobactam; FDC = cefiderocol; S = susceptible; I = susceptible, increased exposure; R = resistant.

## Data Availability

The original contributions presented in this study are included in the article/Supplementary Materials. Further inquiries can be directed to the corresponding author.

## Supplementary Materials

The following supporting information can be downloaded at https://www.mdpi.com/article/10.3390/jcm15051683/s1, Table S1: Virulome genes detected in CRPA invasive and colonizing clinical strains.

## Abbreviations

The following abbreviations are used in this manuscript:

CRPA	Carbapenem-resistant *Pseudomonas aeruginosa*
WGS	Whole genome sequencing
NGS	Next generation sequencing
MLST	Multi-locus sequence typing
ARG-ANNOT	Antibiotic Resistance Gene-ANNOTation
CARD	Comprehensive Antibiotic Resistance Database
VFD	Virulence Factor Database
WHO	World Health Organization
HAIs	Healthcare-associated infections
COPD	Chronic obstructive pulmonary disease
VAP	Ventilator-associated pneumonia
CF	Cystic fibrosis
MDR	Multidrug-resistant
STs	Sequence-based typing
MEM	Meropenem
ARDS	Acute respiratory distress syndrome
BAS	Bronchial aspirate
BAL	Bronchoalveolar lavage
TS	Throat swabs
RS	Rectal swabs
WS	Wound swabs
MALDI-TOF MS	Matrix-assisted laser desorption/ionization time-of-flight mass spectrometry
AST	Antimicrobial susceptibility testing
AK	Amikacin
FEP	Cefepime
CS	Colistin
TZP	Piperacillin–tazobactam
IMR	Imipenem–relebactam
MEV	Meropenem–vaborbactam
CAZ-AVI	ceftazidime–avibactam
C/T	Ceftolozane–tazobactam
FDC	Cefiderocol
EUCAST	European Committee on Antimicrobial Susceptibility Testing
ATB	Antimicrobial therapy
XDR	Extensively drug-resistant
