# Supplementary material for: Genomic Insights into Carbapenem-Resistant Pseudomonas aeruginosa (CRPA): Resistome and Virulome Analysis Beyond Carbapenemases
Source: J Clin Med. 2026 Feb 24;15(5):1683. doi: 10.3390/jcm15051683 (PMC12985536; doi:10.3390/jcm15051683)
Supplement: Supplementary file 1 [file jcm-15-01683-s001.zip › jcm-4102764-supplementary.pdf]

Table S1. Virulome genes detected in CRPA invasive and colonizing clinical strains

		number of strains (%) carrying virulome genes for each group	
**Gene**	**Function**	**Colonizing**	**Invasive**
alg44	Alginate biosynthesis protein Alg44	12 (100)	14 (100)
alg8	Alginate biosynthesis protein Alg8	12 (100)	14 (100)
algA	Phosphomannose isomerase / guanosine 5'-diphospho-D-mannose pyrophosphorylase	12 (100)	14 (100)
algB	Two-component response regulator AlgB	12 (100)	14 (100)
algC	Phosphomannomutase AlgC	12 (100)	14 (100)
algD	GDP-mannose 6-dehydrogenase AlgD	12 (100)	14 (100)
algE	Alginate production outer membrane protein AlgE precursor	12 (100)	14 (100)
algF	Alginate o-acetyltransferase AlgF	12 (100)	14 (100)
algG	Alginate-c5-mannuronan-epimerase AlgG	12 (100)	14 (100)
algI	Alginate o-acetyltransferase AlgI	12 (100)	14 (100)
algJ	Alginate o-acetyltransferase AlgJ	12 (100)	14 (100)
algK	Alginate biosynthetic protein AlgK precursor	12 (100)	14 (100)
algL	Poly(beta-d-mannuronate) lyase precursor AlgL	12 (100)	14 (100)
algP_algR3	Alginate regulatory protein AlgP	12 (100)	14 (100)
algQ	Alginate regulatory protein AlgQ	12 (100)	14 (100)
algR	Alginate biosynthesis regulatory protein AlgR	12 (100)	14 (100)
algU	Sigma factor AlgU	12 (100)	14 (100)
algW	Membrane-bound periplasmic serine protease	12 (100)	14 (100)
algX	Alginate biosynthesis protein AlgX	12 (100)	14 (100)
algZ	Alginate and motility regulator Z	12 (100)	14 (100)
aprA	Alkaline metalloproteinase	12 (100)	14 (100)
asnB	Asparagine synthetase	1 (8,3)	1 (7,1)
cheY	Chemotaxis protein	12 (100)	14 (100)
cheZ	Chemotaxis protein	12 (100)	14 (100)
chpA	Chemotaxis protein	12 (100)	14 (100)
chpB	Probable methylesterase	12 (100)	14 (100)
chpC	Probable chemotaxis protein	12 (100)	14 (100)
chpD	Probable transcriptional regulator	12 (100)	14 (100)
chpE	Probable chemotaxis protein	12 (100)	14 (100)
clpV1	Component of active type VI secretion system (T6SS)	12 (100)	14 (100)
crc	Catabolite repression control protein	12 (100)	14 (100)

cupA1	Fimbrial biogenesis	12 (100)	14 (100)
cupA2	Fimbrial biogenesis	12 (100)	14 (100)
cupA3	Fimbrial biogenesis	12 (100)	14 (100)
cupA4	Fimbrial biogenesis	11 (92)	13 (93)
cupA5	Fimbrial biogenesis	12 (100)	14 (100)
cupB1	Fimbrial biogenesis	12 (100)	14 (100)
cupB2	Fimbrial biogenesis	12 (100)	14 (100)
cupB3	Fimbrial biogenesis	8 (67)	9 (64,3)
cupB4	Fimbrial biogenesis	12 (100)	14 (100)
cupB5	Fimbrial biogenesis	12 (100)	14 (100)
cupB6	Fimbrial biogenesis	10 (84)	12 (86)
cupC1	Fimbrial biogenesis	8 (67)	8 (57,1)
cupC2	Fimbrial biogenesis	12 (100)	14 (100)
cupC3	Fimbrial biogenesis	12 (100)	14 (100)
dotU1	type VI secretion system protein	12 (100)	14 (100)
estA	Esterase	12 (100)	14 (100)
exoS	Exotoxin	5 (42)	7 (50)
exoT	Exotoxin	11 (92)	11 (79)
exoU	Exotoxin	7 (58,3)	7 (50)
exoY	Exotoxin	10 (83,3)	12 (86)
exsA	Transcriptional regulator	12 (100)	14 (100)
exsB	Exoenzyme	12 (100)	14 (100)
exsC	Exoenzyme	12 (100)	14 (100)
exsD	Anti-activator protein	12 (100)	14 (100)
exsE	Secreted regulator of type III secretion genes	12 (100)	14 (100)
fapA	Component of the fibril functional amyloid	12 (100)	14 (100)
fapB	Component of the fibril functional amyloid	12 (100)	14 (100)
fapC	Component of the fibril functional amyloid	12 (100)	14 (100)
fapD	Component of the fibril functional amyloid	12 (100)	14 (100)
fapE	Component of the fibril functional amyloid	12 (100)	14 (100)
fapF	Component of the fibril functional amyloid	12 (100)	14 (100)
fha1	Activation factor of T6S	12 (100)	14 (100)
fimL	Biofilm formation	12 (100)	14 (100)
fimT	Type 4 fimbrial biogenesis protein	10 (83,3)	13 (93)
fimU	Type 4 fimbrial biogenesis protein	12 (100)	14 (100)
fimV	Motility protein	12 (100)	14 (100)
fimX	Phosphodiesterase	12 (100)	14 (100)

fleN	Flagellar synthesis regulator	12 (100)	14 (100)
fleP_fliT	Flagellar attachment protein	4 (33,3)	4 (29)
fleQ	Transcriptional regulator	12 (100)	14 (100)
fleR	Two-component response regulator	12 (100)	14 (100)
fleS	Two-component response regulator	12 (100)	14 (100)
flgA	Flagellar basal body P-ring biosynthesis protein	12 (100)	14 (100)
flgB	Flagellar basal-body rod protein	12 (100)	14 (100)
flgC	Flagellar basal-body rod protein	12 (100)	14 (100)
flgD	Flagellar basal-body rod modification protein	12 (100)	14 (100)
flgE	Flagellar hook protein	12 (100)	14 (100)
flgF	Flagellar basal-body rod protein	12 (100)	14 (100)
flgG	Flagellar basal-body rod protein	12 (100)	14 (100)
flgH	Flagellar L-ring protein precursor	12 (100)	14 (100)
flgI	Flagellar P-ring protein precursor	12 (100)	14 (100)
flgJ	Flagellar protein	12 (100)	14 (100)
flgK	Flagellar hook-associated protein 1	4 (33,3)	4 (29)
flgL	Flagellar hook-associated protein type 3	4 (33,3)	5 (36)
flgM	Flagerllar protein	12 (100)	14 (100)
flgN	Flagellar export chaperone	12 (100)	14 (100)
flhA	Flagellar biosynthesis protein	12 (100)	14 (100)
flhB	Flagellar biosynthetic protein FlhB	12 (100)	14 (100)
flhF	Flagellar biosynthesis protein FlhF	12 (100)	14 (100)
fliA	Sigma factor FliA	12 (100)	14 (100)
fliC	Flagellin type B	12 (100)	13 (93)
fliD	Flagellar capping protein FliD	4 (33,3)	4 (29)
fliE	Flagellar hook-basal body complex protein FliE	12 (100)	14 (100)
fliF	Flagella M-ring outer membrane protein precursor	12 (100)	14 (100)
fliG	Flagellar motor switch protein FliG	12 (100)	14 (100)
fliH	Probable flagellar assembly protein	12 (100)	14 (100)
fliI	Flagellum-specific ATP synthase FliI	12 (100)	14 (100)
fliJ	Flagellar protein FliJ	12 (100)	14 (100)
fliK	Flagellar protein	12 (100)	14 (100)
fliL	Flagellar protein	12 (100)	14 (100)
fliM	Flagellar motor switch protein FliM	12 (100)	14 (100)
fliN	Flagellar motor switch protein FliN	12 (100)	14 (100)
fliO	Flagellar protein FliO	12 (100)	14 (100)

fliP	Flagellar biosynthetic protein FliP	12 (100)	14 (100)
fliQ	Flagellar biosynthetic protein FliQ	12 (100)	14 (100)
fliR	Flagellar biosynthetic protein FliR	10 (83,3)	11 (79)
fliS	Flagellar protein	4 (33,3)	4 (29)
flp	Type IVb pilin	4 (33,3)	3 (21,4)
fppA	Prepilin peptidase	12 (100)	14 (100)
fptA	Ferripyochelin receptor	12 (100)	14 (100)
fpvA	Ferripyoverdine receptor	9 (75)	14 (100)
fpvI	Responsible for transcription of pyoverdine biosynthetic genes	5 (42)	9 (64,3)
fpvR	Probable anti-sigma factor	5 (42)	9 (64,3)
gacA	Component of regulatory system	12 (100)	14 (100)
gacS	Component of regulatory system	12 (100)	14 (100)
hasAp	Heme acquisition protein	12 (100)	14 (100)
hasD	Transport protein HasD	11 (92)	13 (93)
hasE	Metalloprotease secretion protein	12 (100)	14 (100)
hasR	Heme uptake outer membrane receptor	12 (100)	14 (100)
hcnA	Hydrogen cyanide synthase	12 (100)	14 (100)
hcnB	Hydrogen cyanide synthase	12 (100)	14 (100)
hcnC	Hydrogen cyanide synthase	12 (100)	14 (100)
hcp1	Type VI secretion system substrate	12 (100)	14 (100)
hcpA	Secreted protein	12 (100)	14 (100)
hdtS	Not yet reported in current literature data	12 (100)	14 (100)
hisF2	Imidazoleglycerol-phosphate synthase	0	1 (7,1)
hisH2	Glutamine amidotransferase	0	1 (7,1)
hsiA1	Type VI secretion system hcp secretion island protein	12 (100)	14 (100)
hsiB1_vipA_tssB	Type VI secretion system tubule-forming protein	12 (100)	14 (100)
hsiC1_vipB_tssC	Type VI secretion system tubule-forming protein	12 (100)	14 (100)
hsiE1	Type VI secretion system hcp secretion island protein	12 (100)	14 (100)
hsiF1_tssE	Type VI secretion system hcp secretion island protein	12 (100)	14 (100)
hsiG1_tssF	Type VI secretion system hcp secretion island protein	12 (100)	14 (100)
hsiH1_tssG	Type VI secretion system hcp secretion island protein	12 (100)	14 (100)
hsiJ1	Type VI secretion system hcp secretion island protein	12 (100)	14 (100)
icmF1_tssM1	type VI secretion system protein	12 (100)	14 (100)
lasA	LasA protease precursor	9 (75)	13 (93)
lasB	Elastase	12 (100)	14 (100)
lasI	Autoinducer synthesis protein LasI	12 (100)	14 (100)
lasR	Transcriptional regulator LasR	11 (92)	13 (93)

lip1	Outer membrane lipoprotein	12 (100)	14 (100)
mbtH-like	MbtH-like protein from the pyoverdine cluster	12 (100)	14 (100)
motA	Chemotaxis protein MotA	12 (100)	14 (100)
motB	Chemotaxis protein MotB	12 (100)	14 (100)
motC	Flagellar motor protein	12 (100)	14 (100)
motD	Flagellar motor protein	12 (100)	14 (100)
motY	Probable outer membrane protein precursor	12 (100)	14 (100)
mucA	Anti-sigma factor MucA	12 (100)	14 (100)
mucB	Negative regulator for alginate biosynthesis MucB	12 (100)	14 (100)
mucC	Positive regulator for alginate biosynthesis MucC	12 (100)	14 (100)
mucD	Serine protease MucD precursor	12 (100)	14 (100)
mucE	Mucoid induction factor MucE	12 (100)	14 (100)
mucP	Positive alginate regulator MucP	12 (100)	14 (100)
pchA	Salicylate biosynthesis isochorismate synthase	12 (100)	14 (100)
pchB	Salicylate biosynthesis protein PchB	12 (100)	14 (100)
pchC	Pyochelin biosynthetic protein PchC	12 (100)	14 (100)
pchD	Pyochelin biosynthesis protein PchD	12 (100)	14 (100)
pchE	Dihydroaeruginoic acid synthetase	12 (100)	14 (100)
pchF	Pyochelin synthetase	12 (100)	14 (100)
pchG	Pyochelin biosynthetic protein PchG	12 (100)	14 (100)
pchH	Probable ATP-binding component of ABC transporter	12 (100)	14 (100)
pchI	Probable ATP-binding component of ABC transporter	12 (100)	14 (100)
pchR	Transcriptional regulator PchR	12 (100)	14 (100)
pcr1	type III secretion system protein Pcr1	12 (100)	14 (100)
pcr2	type III secretion system protein Pcr2	12 (100)	14 (100)
pcr3	type III secretion system protein Pcr3	12 (100)	14 (100)
pcr4	type III secretion system protein Pcr4	12 (100)	14 (100)
pcrD	Type III secretory apparatus Protein PcrD	12 (100)	14 (100)
pcrG	Regulator in type III secretion	12 (100)	14 (100)
pcrH	Regulatory protein PcrH	12 (100)	14 (100)
pcrR	Transcriptional regulator protein PcrR	12 (100)	14 (100)
pcrV	Type III secretion protein PcrV	12 (100)	14 (100)
phuR	Heme/Hemoglobin uptake outer membrane receptor PhuR precursor	12 (100)	14 (100)
phuT	Heme-transport protein, PhuT	12 (100)	14 (100)
phzA1	Probable phenazine biosynthesis protein	12 (100)	14 (100)
phzB1	Probable phenazine biosynthesis protein	12 (100)	14 (100)
phzB2	Probable phenazine biosynthesis protein	12 (100)	14 (100)

phzC1	Phenazine biosynthesis protein PhzC	12 (100)	14 (100)
phzD1	Phenazine biosynthesis protein PhzD	12 (100)	14 (100)
phzE1	Phenazine biosynthesis protein PhzE	12 (100)	14 (100)
phzF1	Probable phenazine biosynthesis protein	12 (100)	14 (100)
phzG1	Probable pyridoxamine 5'-phosphate oxidase	12 (100)	14 (100)
phzH	Potential phenazine-modifying enzyme	12 (100)	14 (100)
phzM	Probable phenazine-specific methyltransferase	12 (100)	14 (100)
phzS	Flavin-containing monooxygenase	12 (100)	14 (100)
pilA	Type 4 fimbrial precursor	2 (17)	1 (7,1)
pilB	Type 4 fimbrial biogenesis protein PilB	12 (100)	14 (100)
pilC	Type 4 fimbrial biogenesis protein PilC	3 (25)	10 (71,4)
pilE	Type 4 fimbrial biogenesis protein PilE	12 (100)	14 (100)
pilF	Type 4 fimbrial biogenesis protein PilF	12 (100)	14 (100)
pilG	Twitching motility protein PilG	12 (100)	14 (100)
pilH	Twitching motility protein PilH	10 (83,3)	13 (93)
pilI	Twitching motility protein PilI	12 (100)	14 (100)
pilJ	Twitching motility protein PilJ	12 (100)	14 (100)
pilK	Methyltransferase PilK	12 (100)	14 (100)
pilM	Type 4 fimbrial inner membrane platform protein PilM	12 (100)	14 (100)
pilN	Type 4 fimbrial biogenesis protein PilN	12 (100)	14 (100)
pilO	Type 4 fimbrial biogenesis protein PilO	12 (100)	14 (100)
pilP	Type 4 fimbrial biogenesis protein PilP	12 (100)	14 (100)
pilQ	Type 4 fimbrial biogenesis outer membrane protein PilQ precursor	12 (100)	14 (100)
pilR	Two-component response regulator PilR	12 (100)	14 (100)
pilS	Two-component sensor PilS	12 (100)	14 (100)
pilT	Twitching motility protein PilT	12 (100)	14 (100)
pilU	Twitching motility protein PilU	12 (100)	14 (100)
pilV	Type 4 fimbrial biogenesis protein PilV	10 (83,3)	13 (93)
pilW	Type 4 fimbrial biogenesis protein PilW	12 (100)	14 (100)
pilX	Type 4 fimbrial biogenesis protein PilX	12 (100)	14 (100)
pilY1	Type 4 fimbrial biogenesis protein PilY1	11 (92)	9 (64,3)
pilY2	Type 4 fimbrial biogenesis protein PilY2	10 (83,3)	13 (93)
pilZ	Type 4 fimbrial biogenesis protein PilZ	12 (100)	14 (100)
plcB	Phospholipase C, PlcB	12 (100)	14 (100)
plcH	Hemolytic phospholipase C precursor	12 (100)	14 (100)
plcN	Non-hemolytic phospholipase C precursor	12 (100)	14 (100)
pldA	Phospholipase D effector	9 (75)	8 (57,1)

popB	Translocator protein PopB	12 (100)	14 (100)
popD	Translocator outer membrane protein PopD precursor	12 (100)	14 (100)
popN	Type III secretion outer membrane protein PopN precursor	12 (100)	14 (100)
ppkA	Serine/threonine protein kinase PpkA	12 (100)	14 (100)
pppA	Pseudomonas protein phosphatase PppA	12 (100)	14 (100)
pprA	Two-component sensor PprA	12 (100)	14 (100)
pprB	Two-component response regulator, PprB	12 (100)	14 (100)
prpL	Protease IV	12 (100)	14 (100)
pscB	Type III export apparatus protein	12 (100)	14 (100)
pscC	Type III secretion outer membrane protein PscC precursor	12 (100)	14 (100)
pscD	Type III export protein PscD	12 (100)	14 (100)
pscE	Type III export protein PscE	12 (100)	14 (100)
pscF	Type III export protein PscF	12 (100)	14 (100)
pscG	Type III export protein PscG	12 (100)	14 (100)
pscH	Type III export protein PscH	12 (100)	14 (100)
pscI	Type III export protein PscI	12 (100)	14 (100)
pscJ	Type III export protein PscJ	12 (100)	14 (100)
pscK	Type III export protein PscK	12 (100)	14 (100)
pscL	Type III export protein PscL	12 (100)	14 (100)
pscN	ATP synthase in type III secretion system	12 (100)	14 (100)
pscO	Translocation protein in type III secretion	12 (100)	14 (100)
pscP	Translocation protein in type III secretion	12 (100)	14 (100)
pscQ	Translocation protein in type III secretion	12 (100)	14 (100)
pscR	Translocation protein in type III secretion	12 (100)	14 (100)
pscS	Probable translocation protein in type III secretion	12 (100)	14 (100)
pscT	Translocation protein in type III secretion	12 (100)	14 (100)
pscU	Translocation protein in type III secretion	12 (100)	14 (100)
ptxR	Transcriptional regulator PtxR	12 (100)	14 (100)
pvcA	Paerucumarin biosynthesis protein PvcA	12 (100)	14 (100)
pvcB	Paerucumarin biosynthesis protein PvcB	12 (100)	14 (100)
pvcC	Paerucumarin biosynthesis protein PvcC	12 (100)	14 (100)
pvcD	Paerucumarin biosynthesis protein PvcD	12 (100)	14 (100)
pvdA	L-ornithine N5-oxygenase	11 (92)	14 (100)
pvdD	Pyoverdine synthetase D	5 (42)	8 (57,1)
pvdE	Pyoverdine biosynthesis protein PvdE	12 (100)	14 (100)
pvdF	Pyoverdine synthetase F	12 (100)	14 (100)
pvdG	Pyoverdine biosynthesis protein PvdG	12 (100)	14 (100)

pvdH	L-2,4-diaminobutyrate:2-ketoglutarate 4-aminotransferase, PvdH	12 (100)	14 (100)
pvdI	Pyoverdine peptide synthetase	12 (100)	13 (93)
pvdJ	Pyochelin and pyoverdine synthesis	6 (50)	11 (79)
pvdL	Peptide synthase PvdL	11 (92)	14 (100)
pvdM	Dipeptidase precursor	8 (67)	11 (79)
pvdN	Periplasmic aminotransferase PvdN	11 (92)	13 (93)
pvdO	Pyoverdine biosynthesis protein PvdO	10 (83,3)	12 (86)
pvdP	Tyrosinase required for pyoverdine maturation	12 (100)	14 (100)
pvdQ	3-oxo-C12-homoserine lactone acylase PvdQ	12 (100)	14 (100)
pvdS	Sigma factor PvdS	4 (33,3)	9 (64,3)
pvdY	Acetylation of hydroxyornithine	6 (50)	13 (93)
rcpA	Fimbrial biogenesis	12 (100)	14 (100)
rcpC	Fimbrial biogenesis	12 (100)	14 (100)
rhlA	Rhamnosyltransferase chain A	12 (100)	14 (100)
rhlB	Rhamnosyltransferase chain B	12 (100)	14 (100)
rhlC	Rhamnosyltransferase 3	12 (100)	14 (100)
rhlI	Autoinducer synthesis protein RhlI	11 (92)	14 (100)
rhlR	Transcriptional regulator RhlR	11 (92)	13 (93)
rpoN	RNA polymerase sigma-54 factor	12 (100)	14 (100)
rpoS	Sigma factor RpoS	12 (100)	14 (100)
spcS	Specific Pseudomonas chaperone for ExoS, SpcS	12 (100)	14 (100)
spcU	Chaperone required for efficient secretion of the ExoU cytotoxin	7 (58,33)	7 (50)
stk1	Ser/Thr Protein Kinase	12 (100)	14 (100)
stp1	Ser/Thr phosphatase	12 (100)	14 (100)
tadA	TadA ATPase	12 (100)	14 (100)
tadB	component of the Flp/Tad (tight adherence) pilus assembly system	12 (100)	14 (100)
tadC	component of the Flp/Tad (tight adherence) pilus assembly system	12 (100)	14 (100)
tadD	component of the Flp/Tad (tight adherence) pilus assembly system	12 (100)	14 (100)
tadG	component of the Flp/Tad (tight adherence) pilus assembly system	12 (100)	14 (100)
tadZ	component of the Flp/Tad (tight adherence) pilus assembly system	12 (100)	14 (100)
tagF_pppB	Type VI secretion system-associated protein TagF	12 (100)	14 (100)
tagQ	Type VI secretion system-associated lipoprotein TagQ	12 (100)	14 (100)
tagR	Type VI secretion system-associated regulator TagR	12 (100)	14 (100)
tagS	Type IV secretion associated ABC transporter permease TagS	12 (100)	14 (100)
tagT	Type IV secretion associated ABC transporter ATP-binding protein TagT	12 (100)	14 (100)
toxA	Exotoxin A precursor	12 (100)	14 (100)
tse1	Type VI secretion system effector peptidoglycanhydrolase Tse2	12 (100)	14 (100)

tse2	Type VI secretion system effector Tse3	12 (100)	14 (100)
tse3	Type VI secretion system<br>effector muramidase Tse4	12 (100)	14 (100)
tse7	Type VI secretion system effector Dnase Tse8	7 (58,3)	5 (36)
vfr	Transcriptional regulator Vfr	12 (100)	14 (100)
vgrG1a	Type VI secretion system spike protein VgrG1a	12 (100)	14 (100)
vgrG1b	Type VI secretion system spike protein VgrG1b	12 (100)	14 (100)
waaA	3-deoxy-D-manno-octulosonic-acid (KDO) transferase	12 (100)	14 (100)
waaC	Heptosyltransferase I	12 (100)	14 (100)
waaF	Heptosyltransferase II	12 (100)	14 (100)
waaG	UDP-glucose:(heptosyl) LPS alpha 1,3-glucosyltransferase WaaG	12 (100)	14 (100)
waaP	Lipopolysaccharide kinase WaaP	12 (100)	14 (100)
wbpA	UDP-N-acetyl-d-glucosamine 6-Dehydrogenase	0	1 (7,1)
wbpB	UDP-2-acetamido-2-deoxy-d-glucuronic acid 3-dehydrogenase	0	1 (7,1)
wbpD	UDP-2-acetamido-3-amino-2,3-dideoxy-d-glucuronic acid N-acetyltransferase	0	1 (7,1)
wbpE	UDP-2-acetamido-2-dideoxy-d-ribo-hex-3-uluronic acid transaminase	0	1 (7,1)
wbpG	LPS biosynthesis protein	0	1 (7,1)
wbpH	Probable glycosyltransferase	0	1 (7,1)
wbpI	UDP-N-acetylglucosamine 2-epimerase	0	1 (7,1)
wbpJ	Glycosyl transferase	0	1 (7,1)
wbpK	Probable NAD-dependent epimerase/dehydratase	0	1 (7,1)
wbpL	Glycosyltransferase	0	1 (7,1)
wecB	UDP-N-acetylglucosamine 2-epimerase (non-hydrolyzing)	0	2 (14,3)
wzx	O-antigen traslocase	0	1 (7,1)
wzy	B-band O-antigen polymerase	0	1 (7,1)
wzz	O-antigen chain length regulator	0	1 (7,1)
xcpA_pilD	Type 4 prepilin peptidase PilD	10 (83,3)	10 (71,4)
xcpP	Secretion protein XcpP	12 (100)	14 (100)
xcpQ	General secretion pathway protein D	8 (67)	10 (71,4)
xcpR	General secretion pathway protein E	12 (100)	14 (100)
xcpS	General secretion pathway protein F	12 (100)	14 (100)
xcpT	General secretion pathway protein G	12 (100)	14 (100)
xcpU	General secretion pathway outer membrane protein H precursor	12 (100)	14 (100)
xcpV	General secretion pathway protein I	12 (100)	14 (100)
xcpW	General secretion pathway protein J	12 (100)	14 (100)
xcpX	General secretion pathway protein K	12 (100)	14 (100)
xcpY	General secretion pathway protein L	12 (100)	14 (100)

xcpZ	General secretion pathway protein M	12 (100)	14 (100)
